# Subclinical infection caused by a recombinant vaccine-like strain poses high risks of lumpy skin disease virus transmission

**DOI:** 10.3389/fvets.2024.1330657

**Published:** 2024-04-02

**Authors:** Irina Shumilova, Pavel Prutnikov, Ali Mazloum, Alena Krotova, Nikita Tenitilov, Olga Byadovskaya, Ilya Chvala, Larisa Prokhvatilova, Alexander Sprygin

**Affiliations:** Federal Center for Animal Health, Vladimir, Russia

**Keywords:** lumpy skin disease virus, recombinant vaccine-like viruses, subclinical infection, virus transmission, experiment

## Abstract

Lumpy skin disease (LSD) is a transboundary viral infection, affecting cattle with characteristic manifestations involving multiple body systems. A distinctive characteristic of lumpy skin disease is the subclinical disease manifestation wherein animals have viremia and shed the virus through nasal and ocular discharges, while exhibiting no nodules but enlarged lymph nodes that are easily oversighted by inexperienced vets. Further research on the role of subclinically ill animals in the transmission of LSD virus (LSDV) can contribute to the development of more effective tools to control the disease worldwide. Thus, this study aims to determine the potential role of subclinical infection in virus transmission in a non-vector-borne manner. To achieve this, we inoculated animals with the recombinant vaccine-like strain (RVLS) Udmurtiya/2019 to cause clinical and subclinical LSDV infection. After the disease manifestation, we relocated the subclinically ill animals to a new clean facility followed by the introduction of another five animals to determine the role of RVLS-induced subclinical infection in the virus transmission via direct/indirect contact. After the introduction of the naïve animals to the relocated subclinically ill ones in a shared airspace, two introduced animals contracted the virus (clinically and subclinically), showing symptoms of fever, viremia, and seroconversion in one animal, while three other introduced animals remained healthy and PCR-negative until the end of the study. In general, the findings of this study suggest the importance of considering LSDV subclinical infection as a high-risk condition in disease management and outbreak investigations.

## 1 Introduction

Lumpy skin disease virus (LSDV) is recognized as an important transboundary pathogen whose infection in animals has been associated with considerable losses in affected farms and countries (1). The etiological agent belongs to the *Capripoxvirus* genus along with the sheep pox virus and goat pox virus, which all share approximately 96% identity (2). The animals that are susceptible to the disease include cattle and buffaloes, among others (3). LSD virus (LSDV) has been shown to have a broader host tropism as previously expected. A recent study reported the isolation of LSDV genomic DNA from the nodules of springboks, oryxes, and giraffes. Research evidence has also confirmed that experimental infection can lead to clinical signs in impalas and giraffes (3–5).

As a species, LSDV has emerged 500 years ago via recombination (6, 7) but was officially first documented in Zambia in 1929, from where it spread throughout Africa and then into the Middle East (8–10).

In the recent decade, LSDV dramatically spread across Eurasia and Southeast Asia (11–13). Unprecedentedly, the LSDV epidemiology in Eurasia was accompanied by the emergence of novel vaccine-like strains that cause homologous recombination between two vaccine strains, i.e., the commercial Neethling vaccine strain and Kenyan KSGP strain used in Lumpivax vaccine (KEVEVAPI) (14, 15). The incidence of RVLS has been increasing in several countries, including China, Thailand, and Mongolia (16–18). In India and Bangladesh, LSDV outbreaks have been attributed to the KSGP strain lineage (19, 20).

Phylogenetically, the current genetic clustering of existing LSDV lineages is divided into the following clusters: (1) Cluster 1.1, which includes vaccine Neethling strains, and (2) Cluster 1.2, which includes classical field strains, such as Warmbaths, Dagestan/2015, Israel, and KSGP-like strains (15, 21). Aside from the classical Cluster 1.1 and 1.2 viruses, newly emerged recombinant strains have been found, which constitute new clusters (from 2.1 to 2.6) (15, 21).

The first recombinant strain Saratov/2017 whose backbone was represented by the Neethling vaccine and KSGP strains was recovered from a field outbreak in 2017 in Russia close to a country that launched a mass vaccination with a live attenuated vaccine against LSDV (14). After the analysis of the Saratov/2017 full genome sequence, this strain comprised novel Cluster 2.1. Another recombinant strain Udmurtia/2019, whose dominant parental strain was the KSGP strain backbone and Neethling vaccine strain as a minor one as opposed to Saratov/2017, belongs to Cluster 2.2, followed by Cluster 2.3 by Kostanay/2018 from Kazakhstan and Cluster 2.4 by Tyumen/2019. The strains from Southeast Asia, especially in China, Thailand, and Vietnam, belong to dominant Cluster 2.5 and have been found to be prominent in the region (11).

LSD can manifest clinically as typical skin nodules and mucosal surface lesions but can also occur in subclinical form without these symptoms. In both cases, viremia and virus shedding through nasal discharges can occur (22–24). Research on these virus shedding sites, regardless of disease manifestation, can help elucidate the role of excreted viruses in transmission to in-contact animals (25–27).

LSDV is known to be mechanically transmitted through arthropod bites only, although the studies that suggested this were only built on Cluster 1.2 strains, while the RVLSs with altered genomes have acquired mechanisms for direct/indirect contact modes of transmission under experimental studies and natural conditions (28–30). Importantly, LSDV transmission without arthropod assistance is commonly observed in recombinant LSDV strains from Cluster 2.1, Cluster 2.2, and the like (31, 32). This feature is critical in LSDV management but is often overlooked due to the lack of awareness and pursuit of the vector-borne concept and limited access to recombinant strains for testing (30, 33). Notably, all capripoxviruses and poxviruses can spread through contact transmission (2). Therefore, considering the capacity of the RVLSs to transmit via direct or indirect contact, subclinical infection caused by the RVLSs can undermine the current efforts of disease prevention. The aim of this study is to investigate the role of RVLS-induced subclinical infection in the non-vector-borne transmission of the virus to in-contact animals.

## 2 Materials and methods

### 2.1 Virus

One RVLS of LSDV was isolated from the Udmurtiya region of the Russian Federation in 2019. This genetic lineage was unique and was never detected anywhere elase (30). This strain was selected for the experiment due to the following criteria: (i) it was detected during snowy winter (30); (ii) the Udmurtiya strain was already shown capable of non vector-borne transmission (31). The virus was isolated by performing two serial passes in goat testis cells before characterization via PCR amplification and the sequencing of several loci specific to either the vaccine or the field strain genome (21, 29). Moreover, the virus was titrated in 96-well plates using tenfold dilution. The plates were incubated at 37°C with 5% CO_2_ for 72 h and inspected daily for the presence of the cytopathic effect (CPE). The virus titer was measured using the Spearman–Karber method as reported previously (31). The results are expressed in logarithm as 50% tissue culture infective dose (log TCID50).

### 2.2 Experimental design

Ten non-vaccinated Russian black pied bulls aged 6–8 months (300–500 kg in weight) were included. Prior to the experiment, the blood and serum were tested for LSDV genome and antibodies to ensure that they had not been exposed to the virus. The animals were numbered from 1 to 10 randomly and housed in an insect-free animal biosafety level 3 facility and subjected to a 12-h light–dark cycle, relative humidity of 30–70%, and temperature range between 23°C and 26°C. Moreover, they were monitored twice a day by the veterinary staff and provided with water and feed *ad libitum*.

All the animals participating in the experiment (a total of 15) were also checked for any presence of ticks before entering the facility and kept in the facility for two weeks before the start of the study for them to adapt to the conditions. Their blood samples and nasal swabs were obtained for PCR and blood for neutralization (NT) tests to exclude previous or present LSDV infections (34, 35).

On the first day of the study (0 dpi), 2 mL of 5 log TCD50/mL of Udmurtiya/2019 virus was used to inoculate each animal intravenously. The animals were monitored daily for skin lumps, whereas the blood samples and nasal swabs were collected every second day for analysis via real-time PCR to detect LSDV nucleic acids. The study and monitoring period lasted for 49 days, which involved the daily registration of body temperature (Supplementary Figure S1) and clinical score (Supplementary Figure S2) based on the recommendations of Wolff et al. (36).

Upon the onset of the first skin lumps, the affected animals were kept in the facility, while the subclinically affected animals (without nodules but with viremia and virus shed via nasal discharge) were transferred to another disinfected room, wherein other five animals (in-contact) were introduced. For the purpose of this study, the term “in-contact animal” pertains to animals that were housed in the same ventilated insect-proof facility sharing airspace where they could see each other, but any physical contact between them as well as sharing of water troughs, food, or bedding were prohibited. Their mobility was also restricted using tethering. Furthermore, the in-contact animals were monitored for clinical signs and viremia via PCR throughout the experiment.

### 2.3 DNA extraction and PCR

The samples were handled aseptically and processed as 10% homogenates in phosphate-buffered saline. A 200-μL aliquot was used for total nucleic acid extraction using the QIAamp DNA Mini Kit (Qiagen, Germany) in accordance with the manufacturer’s instructions. The sample extracts were analyzed to check the presence of LSDV DNA using real-time PCR (qPCR) based on ORF044 as previously described (35).

The fluorogenic probe was labeled at the 5′ end with the FAM reporter dye and BHQ as a quencher at the 3′ end. Selected primers (df4ln: CAAAAACAATCGTAACTAATCCA and zdr4ln: TGGAGTTTTTATGTCATCGTC) and probes (zdpro4ln1:Fam-TCGTCGTCGTTTAAAACTGA-BHQ1) were synthesized by Syntol (Moscow, Russia). PCR was performed using a Rotor-Gene Q (Qiagen, Germany) instrument with the following thermal-cycling profile: 95°C for 10 min, followed by 45 cycles at 95°C for 15 s (s) and 60°C for 60 s. Moreover, the final reaction volume was 25 μL containing 10 pmol of each primer, 5 pmol of the probe, 5 μL of 25 mM MgCl2, 5 μL 5 × PCR buffer (Promega, United States), 1 μL of 10 pmol dNTPs (Invitrogen, USA), and deionized water. The samples were analyzed in accordance with the protocol as previously described (35).

### 2.4 Virus neutralization

Virus neutralization in JetBioFil 96-well flat-bottom microplate (Guangzhou JET Bio-Filtration, China) was conducted in accordance with the protocol previously described (34) with a few modifications. The test was performed on ovine testis cells with two replicates having the same strain Udmurtiya/2019 used as the inoculum. One hundred μl of the virus inoculum was added into each well, and the neutralization dilution was considered positive at <1:8, doubtful at <1:4, and negative at <1:2.

#### 2.4.1 Visualization of results

The data were visualized using Microsoft Excel.

## 3 Results

**Figure 1:**
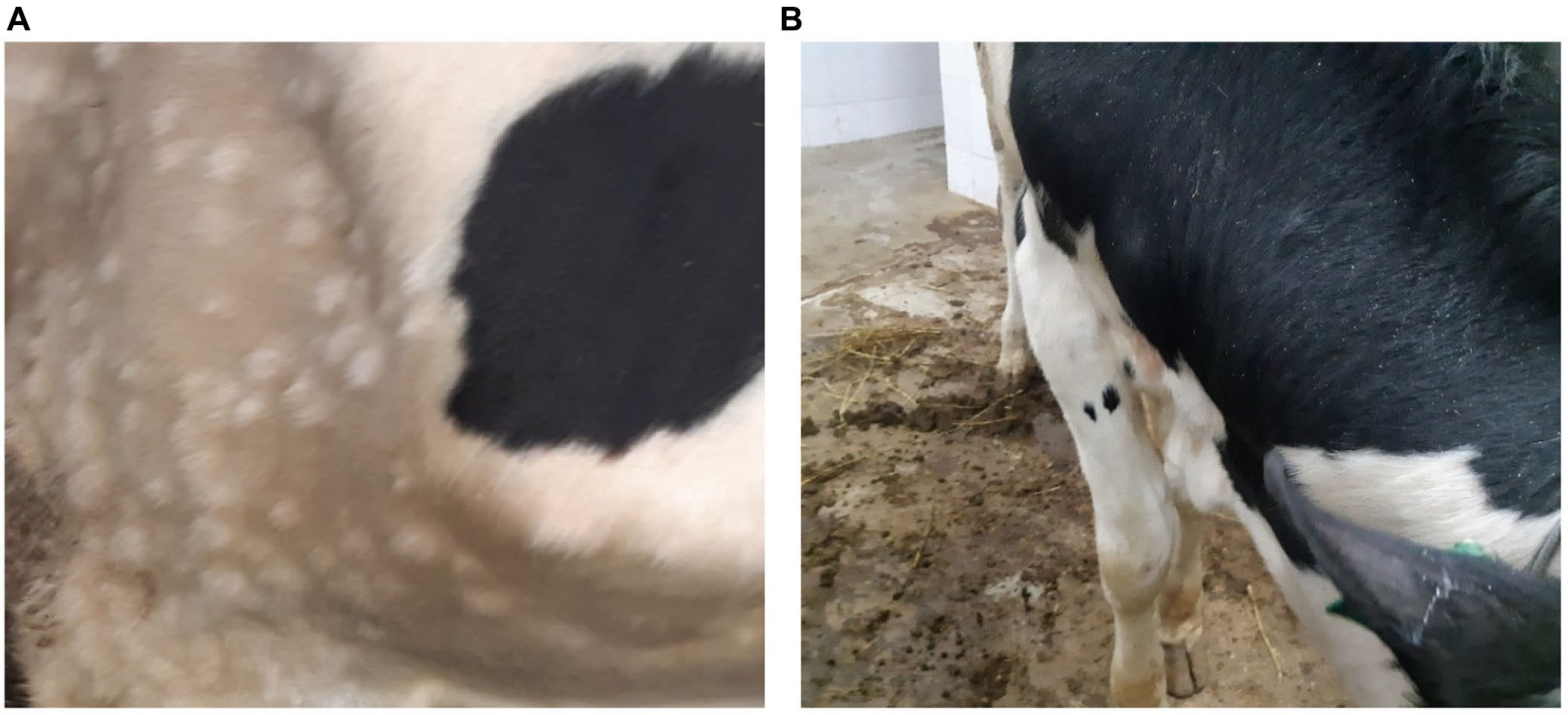
Clinical manifestations of LSDV in the form of nodular skin lesions at 10 dpi. **(A)** Multiple nodular lesions in the scapular region (bull no. 1). **(B)** Nodular lesions on the back (bull no. 4).

The first signs of an increase in body temperature (41.1°C–41.5°C) were recorded in 4 out of 10 infected bulls at 7 dpi (animals 1, 4, 5, and 9). The first clinical manifestations of LSDV were observed at 8–9 dpi in the form of small skin bumps on the neck and shoulder blades (Figure 1).

**Figure 2:**
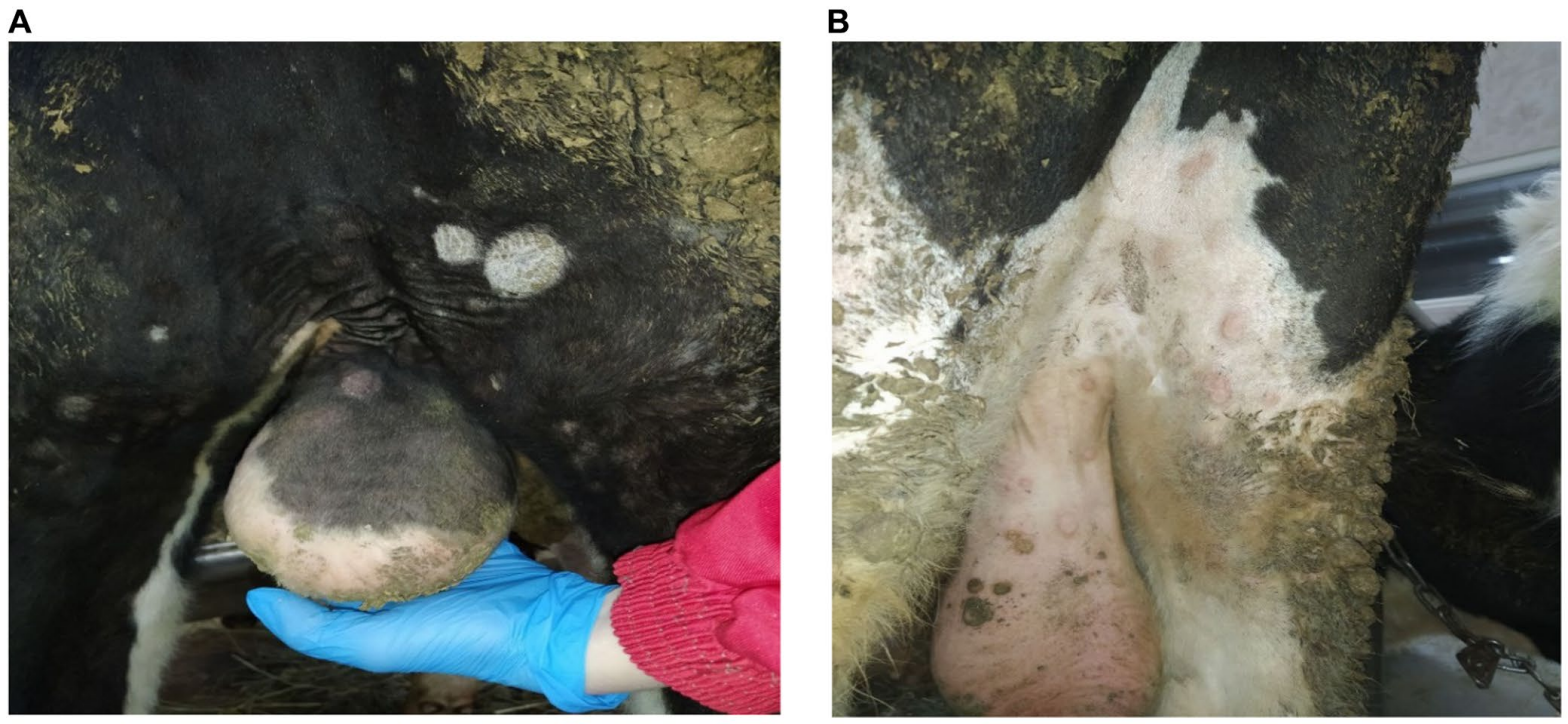
Clinical manifestations of LSDV in the form of roseola at 10 dpi. **(A)** Roseola on the scrotum (bull no. 5). **(B)** Roseola on the scrotum and hindlimbs (bull no. 9).

Aside from roseola on the scrotum (Figure 2), skin lesions ranging in size from 0.3 × 0.3 cm to 2.0 × 2.5 cm were spotted over the entire body surface of these bulls. Moreover, the animals presented with enlarged superficial lymph nodes and signs of increased weakness, heavy breathing, and loss of appetite.

Real-time PCR revealed the LSDV genome in the stabilized blood sample and nasal swabs (Tables 1, 2). Of the 10 inoculated bulls, Bull no. 3 and 8 showed no detectable viremia and shedding (Tables 1, 2). Bull no. 3 and 8 was found to be resistant to LSDV at the end of the experiment. The viremia in the subclinically ill animals lasted for 3–4 days with a Cts ranging from 22.7 to 34.6. Moreover, the nasal shedding lasted for 1–3 days with a Cts varying from 30.1 to 36.1 (Tables 1, 2).

**Table 1:** RT-PCR results of the detection of the LSDV genome in the stabilized blood and swab samples from the 10 virus-inoculated animals.

DPI	Number of anmal	№1		№2		№3		№4		№5		№6		№7		№8		№9		№10	
1–9		–	–	–	–	–	–	–	–	–	–	–	–	–	–	–	–	–	–	–	–
10		27,7		–	–	–	–	–	30,1	–	31,4	22,7	–	–	–	–	–	–	28,9	–	–
12		26,9	32,2	–	–	–	–	27,1	32,5	25,5	29,5	31,2	–	–	–	–	–	25,8	29,3	–	–
14		–	–	–	–	–	–	–	–	–	–	30,8	–	26,6	30,2	–	–	–	–	31,7	–
16		–	–	32,8	31,5	–	–	–	–	–	–	–	–	27,9	29,5	–	–	–	–	34,6	36,1
18		–	–	29,1	30,1	–	–	–	–	–	–	–	–	29,1	28,5	–	–	–	–	34,1	35,4
20		–	–	–	32,2	–	–	–	–	–	–	–	–	–	–	–	–	–	–	26,5	–
22		–	–	–	–	–	–	–	–	–	–	–	–	–	–	–	–	–	–	–	–
24–49		–	–	–	–	–	–	–	–	–	–	–	–	–	–	–	–	–	–	–	–

* –, a negative PCR result, yellow designates Ct for blood, green designates Ct for swabs.

**Table 2:** RT-PCR results of the detection of LSDV genome in the stabilized blood and swab samples from the 5 newly introduced animals.

DPI	Number of anmal	№11		№12		№13		№14		№15	
15–25		–	–	–	–	–	–	–	–	–	–
26		–	–	–	–	30,5	–	–	–	–	–
28		–	–	–	–	32,9	31,5	–	–	–	–
31		–	–	–	–	31,2	30,8	–	–	–	–
33		–	–	–	–	24,8	29,4	24,1	–	–	–
35		–	–	–	–	28,5	32,3	25,7	–	–	–
37		–	–	–	–	30,6	36,7		–	–	–
39–49		–	–	–	–	–	–	–	–	–	–

* –, a negative PCR result, yellow designates Ct for blood, green designates Ct for swabs.

Subsequently, the real-time PCR results for Bull no. 2 and 10 indicated positive values for the blood and nasal swab samples taken at 16 and 14 dpi, respectively (Table 1), although the gross LSDV clinical signs and an increase in body temperature in these two animals were not observed throughout the study.

At 13 dpi, all four bulls (Bull no. 1, 4, 5, and 9) were withdrawn from the study, leaving the remaining animals for further monitoring. On the 15th dpi, the other five healthy bulls (Bull no. 11–15) were introduced and placed between the subclinically infected animals in a new clean facility. Bull no. 7 showed an increase in body temperature to 40.9°C at 16 dpi and presented with signs of depression with a loss of appetite, skin bumps appearing over the entire surface of the body, and an increase in superficial lymph nodes at 18 dpi. Moreover, Bull no. 7 was removed at 19 dpi.

The subclinically ill animals without visible symptoms (Bull no. 2, 3, 6, 8, and 10) and newly introduced animals (Bull no. 11, 12, 13, 14, and 15) remained in the study.

**Figure 3:**
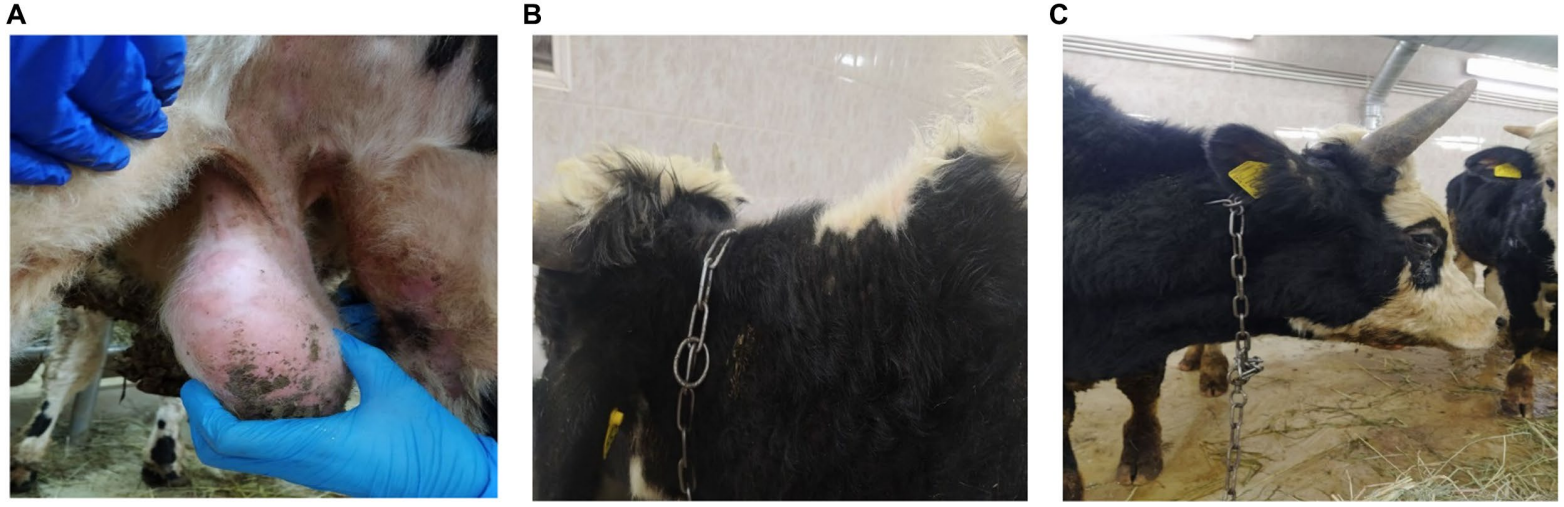
Clinical manifestation of LSDV in Bull no. 13 11 days after contact with the subclinically ill animals. **(A)** Roseola in the scrotum (bull no. 13). **(B)** Multiple nodular lesions (bull no. 13). **(C)** Purulent discharge from the eyes (bull no. 13).

On the 11th day after the introduction of the new animals, Bull no. 13 showed signs of fever with a body temperature of 40.5°C and roseola on the scrotum and the groin at 14 dpi after introduction (Figure 3A). On the 16th day, the animal exhibited skin nodules (Figure 3B), accompanied by a strong cough, an increase in superficial lymph nodes, purulent discharge from the eyes (Figure 3C), and edema of the forelimbs. Moreover, the bull showed symptoms of depression with a loss of appetite and did not get up for 6 days. Fever was maintained for 12 days with a body temperature varying from 40.5°C to 41.5°C.

The NT test results revealed that the subclinically ill animals presented with positive values at the end of the experiment (dpi 49) with a NT dilution ranging from 1:8 to 1:64, whereas the animals that had been removed from the experiment were not analyzed. Bull no. 3 and 8 did not have viremia but had seroconversion (Table 3). Bull no. 11–15 were also sampled for NT analysis, wherein only Bull no. 11 had doubtful results and the others had negative results (< 1:2).

**Table 3:** Neutralization assay results from the 15 animals of the experiment.

Number of animal	№1	№2	№3	№4	№5	№6	№7	№8	№9	№10	№11	№12	№13	№14	№15
NT result	NI	< 1:8	< 1:64	NI	NI	< 1:32	NI	< 1:8	NI	< 1:32	< 1:4	< 1:2	< 1:2	< 1:2	< 1:2

For NT: results < 1:8 are considered positive; < 1:4 doubtful; and < 1:2 negative.

Except for Bull no. 13 (clinical form), no clinical signs were observed in any of the newly introduced animals (Bull no. 11–15) throughout the observation period, although Bull no. 14 showed (subclinical form) only positive PCR results in the blood starting on the 26th day of the study (13 days after the introduction to the infected animals) until the 37th day of the study (Tables 1, 2). Only Bull no. 11 showed doubtful NT results (Table 3).

## 4 Discussion

The transmission of capripoxviruses has been attracting research interest since LSDV was identified in the Northern Hemisphere (37). As in the past, only limited evidence could confirm the contagious nature of LSDV, similar to the situation with sheep pox and goat pox (29), and considering the seasonality of LSDV rebounding (38), efforts were focused on controlling vector-borne transmissions (30, 39). The control of contact transmission based on epidemiological findings without reliable laboratory tools was proposed as early as the onset of LSDV range expansion in Africa (8). Weiss’s hypothesis was supported by recombinant vaccine-like LSDV lineages that have increased in incidence since 2017 following the use of a live attenuated LSDV vaccine, which precipitated the occurrence of the novel RVLSs of LSDV comprised of the Neethling and KSGP vaccine strains (24). Genetically, the RVLSs fall outside the established Cluster 1.1, which was represented by the Neethling strains and virulent Neethling strains circulating in South Africa during the 1990s, and Cluster 1.2, which was represented by strains, such as Warmbaths LW, Dagestan/2015, Bujanovac/2016, Ni-2490, and KSGP (15). Interestingly, it was first observed that the novel recombinant strains spread in a non-vector-borne manner as opposed to the Cluster 1.1 and 1.2 strains (31). Furthermore, it was suggested that Saratov/2017 could spread through a contaminated feed and that the RVLSs acquired a genetic element through recombination that was missing or not overtly expressed from the parental strains (40).

Furthermore, not only the clinical form of LSDV has gained research interest but also the subclinical manifestation has been recognized as a distinctive characteristic of LSDV (41). With the recent emergence of recombinant vaccines, such as strains exhibiting transmission without arthropod activity (31, 32), efforts have been made to determine the contribution of all forms. The studies led by Sprygin et al. analyzed the biological features of recombinant strains, demonstrating that recombinant strains not only show altered properties on cell culture but also overwinter in northern latitudes (31, 40, 42, 43).

The present study follows up on the research on recombinant strains detected in Russia and provides further evidence confirming that the RVLSs of LSDV employ alternative mechanisms of transmission in contrast to that of Cluster 1.2 strains. Moreover, this is the first study to report that subclinically affected animals transmit LSDV to animals sharing the same airspace in an insect-proof facility, which is contrary to the findings of Heageman et al. (41). Subclinical infection is a typical manifestation of LSDV, and the efficiency of its control measures is directly linked to the manner whereby virus-carrying animals, regardless of the clinical or subclinical manifestation of the disease in an outbreak zone, are identified and managed (22, 24, 25). This study contributes to the deeper understanding of LSDV and determine the role of subclinical LSDV infection in LSDV transmission and epidemiology (41).

Our study was designed to induce subclinical infection in bulls with continued monitoring. The subclinical infection in LSDV is accompanied by virus shedding and viremia without obvious clinical signs, which can be overlooked in cattle inspection during suspected or actual outbreak management (23). In the present study, we reproduced the subclinical infection in a laboratory setting, although pure subclinical infection never occurs in a field outbreak, and showed its “contagious nature” by the presence of LSDV DNA. Although the NT analysis of the antibodies revealed doubtful results for one subclinical animal, the findings should be carefully assessed (Tables 1, 2). Nevertheless, subclinical animals concurrently become clinically ill in real field conditions; LSDV caused by the RVLSs also poses a threat of non-vector-borne virus transmission regardless of the disease presentation (40). It is noteworthy that subclinical virus carriers pose a risk of the non-vector-borne transmission of LSDV since they shed LSDV similarly to clinically ill animals (24). Of note, subclinically ill animals shed the virus via excretions (e.g., saliva, snots, ocular fluids), whereas clinically ill animals shed more virus via necrotized and sequestrated nodules (22, 40).

Following the moving of subclinically affected animals that shed LSDV to another disinfected room followed by the placement of naïve animals imitated a natural situation. That resulted in the infection of one animal with clinical signs and fever and one with subclinical infection identified by PCR only with the other three newly introduced animals remaining resistant to the virus. Of note, Bull no. 7 that was removed at 19 dpi (Table 1) could have been interpreted as clinically ill; however, skin bumps or early nodules before necrosis do not shed virus into the environment. Since the virus is entrapped inside them, the swabs from skin bumps/early nodules were negative (data not shown), which should not compromise Bull no. 7 as the source of virus for infecting in-contact animals. Moreover, the testing showed that it was shedding the virus in the same manner with a slightly lower Ct value as the other retained animals through nasal discharge. Thus, it should be determined whether Bull no. 7 could have affected the outcome of the transmission due to the Ct value being 28.5–29.5 compared with that being 30.1–36.1 in subclinical animals by definition (Table 1). Although the LSDV genomes were detected in the blood samples and nasal swabs of Bull no. 13 and 14, the NT results returned negative, which might be associated with the time of performing NT, in which the immune system did not have enough time to produce detectable antibodies (Table 2).

Considering that previous experiments have confirmed the non-vector-borne nature of transmission of the RVLS (24, 31, 40), it is unlikely that the present findings were influenced. In this regard, a quantitative study is needed to address the issue of minimal infective dose for the RVLSs to spread in an air-borne context.

The classification of LSDV-infected animals as clinically ill, subclinically affected, and resistant can be explained by variation in unknown host/genetic factors ranging from resistance to death (39). So far, most animal studies on LSDV have employed strains from Cluster 1.2 comprising unaltered field isolates, whereas a few studies have assessed the properties of the novel RVLSs (22, 33, 44, 45). Weiss hypothesized the implication of contact transmission under field conditions, although molecular techniques were unavailable then (8) and studies under laboratory settings did not report any infection via contact. The RVLSs have gained research interest in relation to their epidemiology, transmission, and diagnostics (2, 46); however, the limited accessibility of the RVLS delays the identification of its properties following the discoveries of novel features that are not observed in parental strains.

Interestingly, Bulls no. 3 and 8 were shown to be resistant to LSDV throughout the experiment but mounted an antibody response by the end of the experiment (Table 1). Although some other animals showed positive PCR results, they showed no seroconversion (Table 1), which is commonly observed during vaccination and experiments (24, 40). Further research on host resistance to LSDV is needed.

Overall, the findings of this study have revealed the transmission risks posed by the RVLSs of LSDV and their resulting subclinical infections that should be primarily investigated in epidemiological studies. Theoretical studies extrapolating from the evidence based on Cluster 1.2 must be limited to the Cluster 1.2 strains. However, if the global LSDV epidemiology is concerned with the Cluster 2.5 strains in Southeast Asia and KSGP-like strains in India, analyses on the Cluster 1.2 strains in the Middle East and Africa should consider the observed epidemiological phenomena inherent to the present-day situation in the field and recombinant strain circulation with their properties, i.e., non-vector-borne transmission. In general, this is the first study to focus primarily on the complicated issues of LSDV transmission, whether it is a classical 1.2 strain lineage or recombinant lineage 2.1 and the like (47). Considering this, further research on subclinical infections is needed to delineate the particular potential of the RVLSs in non-vector-borne virus transmission.

The present study together with published evidence on transmission and recombinant LSDVs emphasizes the importance of LSDV research as well as the re-evaluation of the control and eradication approaches for LSDV (including similar measures applied to sheep pox and goat pox that spread via direct and indirect contact) and recognition of contact transmission, which will inevitably provide a better understanding of the disease, its epidemiological profile and contribute to improved eradication policies.

## Data availability statement

The original contributions presented in the study are included in the article/Supplementary material, further inquiries can be directed to the corresponding author.

## Ethics statement

The animal study was approved by the ethics committee of the Federal Center for Animal Health. The study was conducted in accordance with the local legislation and institutional requirements.

## Author contributions

IS: Investigation, Methodology, Writing – original draft. PP: Conceptualization, Investigation, Methodology, Writing – original draft. AM: Data curation, Writing – original draft, Writing – review & editing. AK: Methodology, Writing – original draft. NT: Methodology, Writing – original draft. OB: Formal analysis, Validation, Writing – original draft, Writing – review & editing. IC: Formal analysis, Project administration, Writing – review & editing. LP: Writing – review & editing. AS: Funding acquisition, Project administration, Resources, Supervision, Visualization, Writing – original draft, Writing – review & editing.

## Supplementary material

### SUPPLEMENTARY TABLE 1

Registered clinical score for each experimental bull based on the recommendations of Wolff et al. (36).
