# Supplementary material for: Subclinical infection caused by a recombinant vaccine-like strain poses high risks of lumpy skin disease virus transmission
Source: Front Vet Sci. 2024 Apr 2;11:1330657. doi: 10.3389/fvets.2024.1330657 (PMC11019024; doi:10.3389/fvets.2024.1330657)
Supplement: Supplementary file 2 [file Image_1.pdf]

Supplementary Materials:

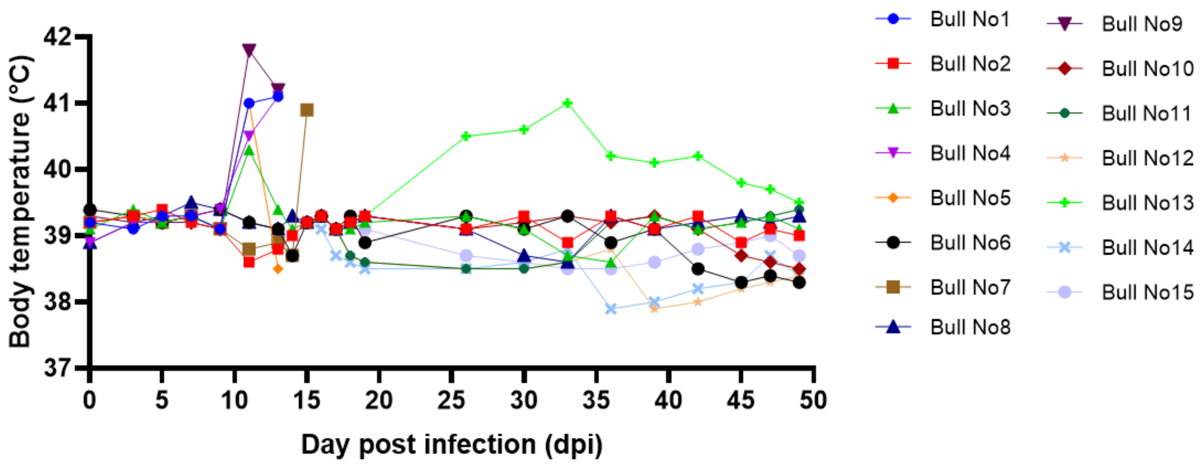

Supplementary Figure 1. Registered body temperature (°C) of the experimental bulls.

An increased body temperature was defined as a body temperature of ≥ 39.6 °C, and fever was defined as starting at a body temperature of ≥ 40.0 °C. This was registered for Bulls no. 1, 3, 4, 7, and 9 (infected group) on the 11th day of the experiment. The body temperature of Bull no. 3 normalized one day later and remained stable for the rest of the experiment, while the other bulls were excluded from the experiment. Bull no. 13 (in-contact group) had an increase in body temperature starting on the 19th dpi and reached 41 °C on the 34th dpi and was above normal until the end of the experiment.

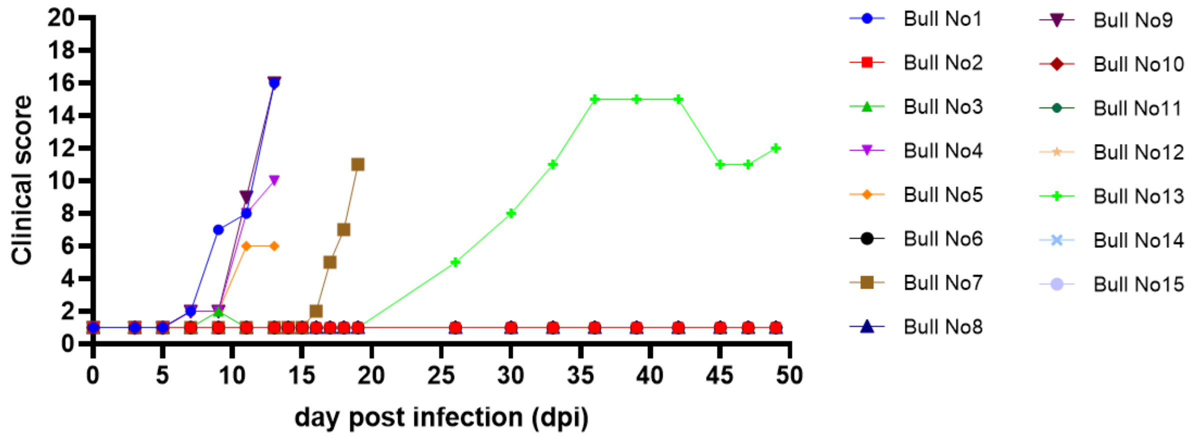

Supplementary Figure 2. Registered clinical score of the experimental bulls.

Based on the recommendations of Wolff et al. (2020), the clinical score is a sum of scores given to specific physiological and behavioral changes () in animals after illness. A normal body temperature has a score of 1, whereas no changes are rated as 0. As seen in the figure, a clinical score of 1 means that the animal showed no clinical changes. The clinical reaction score was determined daily during the animal trial. Human endpoint was set at a clinical score of ≥ 10 or reaching criteria "abandonment."
